# Supplementary material for: The Influence of Motivators and Barriers to Exercise on Attaining Physical Activity and Sedentary Time Guidelines among Canadian Undergraduate Students
Source: Int J Environ Res Public Health. 2022 Sep 27;19(19):12225. doi: 10.3390/ijerph191912225 (PMC9566408; doi:10.3390/ijerph191912225)
Supplement: Supplementary file 1 [file ijerph-19-12225-s001.zip › ijerph-1892647-supplementary.pdf]

Table S1. Distribution of the undergraduate student sample based geographical location of institution by province.

Province/Territory	n (%)
Ontario	104 (30%)
Nova Scotia	66 (19%)
British Columbia	60 (18%)
Alberta	40 (12%)
Manitoba	33 (10%)
New Brunswick	16 (5%)
Newfoundland & Labrador	14 (4%)
Quebec	4 (1%)
Saskatchewan	3 (1%)
Prince Edward Island	1 (<1%)

Table S2. The selection frequency and proportion for the top four motivators and barriers in respondents who self-reported as a varsity athlete, female, obese (body mass index (BMI) ≥ 30 kg•m^{-2}), health student, or enrolled at a West, Central or Atlantic Canadian institution at the time of survey response.

Motivator/Barrier (n)	n (%) of Varsity Athletes	n (%) of Females	n (%) BMI ≥ 30 kg•m^{-2}	n (%) in Health	n (%) in West	n (%) of Central	n (%) in Atlantic
Improved Physical Health (254)	41 (16)	209 (82)	32 (13)	178 (71)	80 (31)	109 (43)	65 (26)
Improved Cognitive/Mental Health (228)	35 (15)	202 (89)	25 (11)	161 (71)	68 (30)	95 (42)	65 (29)
Improved Physical Appearance (203)	26 (13)	172 (85)	26 (13)	69 (34)	54 (27)	90 (44)	59 (29)
Athletic Performance (94)	34 (36)	66 (70)	11 (12)	39 (41)	25 (26)	35 (37)	34 (36)
School Obligations (232)	39 (17)	190 (82)	27 (12)	172 (74)	81 (35)	89 (38)	62 (27)
Time Commitment (197)	36 (18)	161 (82)	27 (14)	132 (67)	64 (32)	83 (42)	50 (25)
Job Obligations (109)	12 (11)	93 (85)	15 (14)	74 (68)	29 (27)	56 (51)	24 (22)
Lack of Fitness Classes (88)	20 (23)	72 (82)	8 (9)	56 (64)	25 (28)	30 (34)	33 (38)

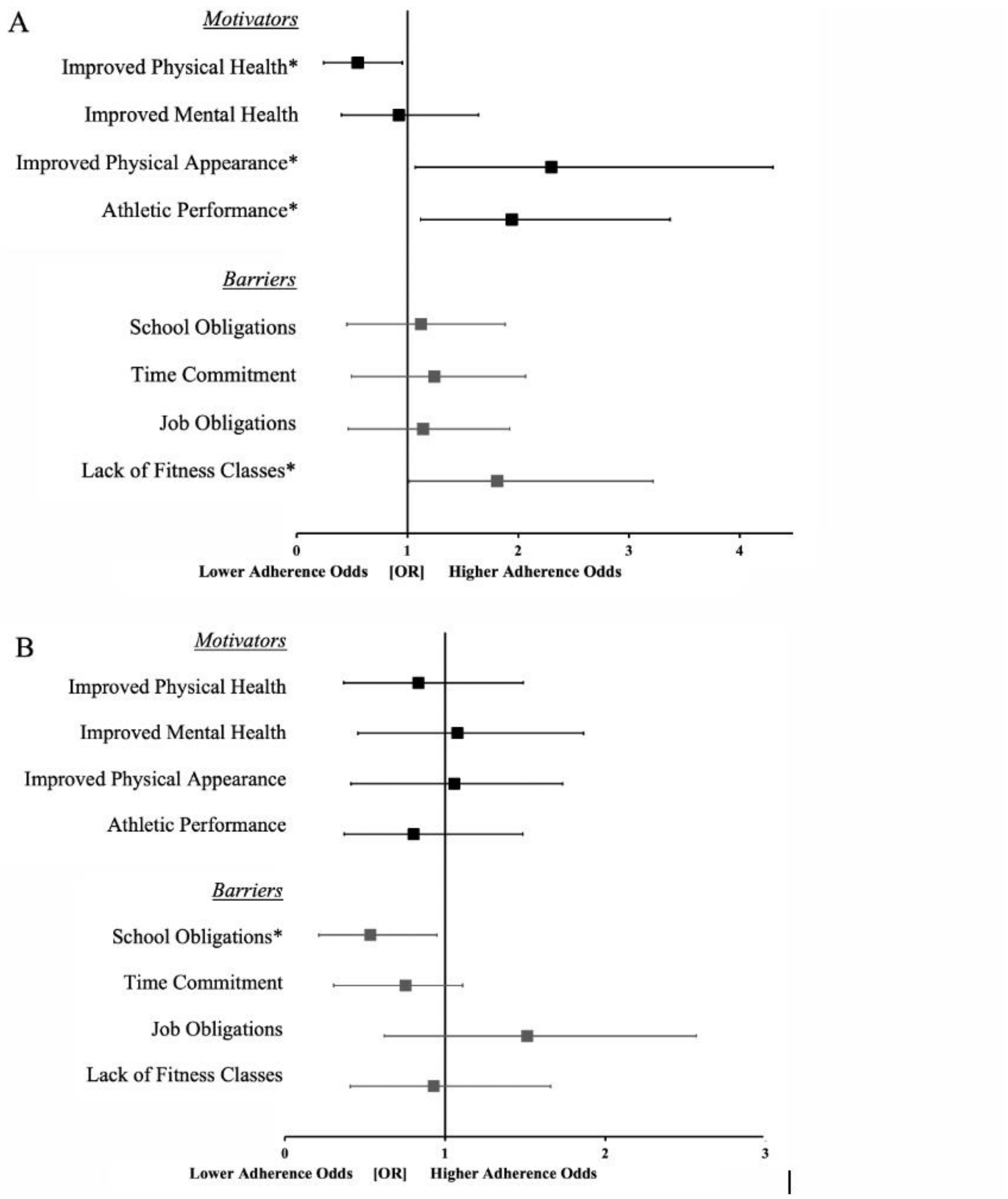

Figure S1. Forest Plot displaying the odds ratio (OR) ± 95% confidence intervals for the motivators and barriers to achieving the physical activity (panel A) and sedentary time (panel B) guidelines in females only. *, indicates a significance of $p < 0.05$. Note: Due to odds ratios having a minimum value of 0 (i.e., cannot be less than 0) and an infinite maximum value, the 95% confidences intervals were asymmetrical in magnitude.
